# Indicación y prescripción de macrólidos en una población colombiana

**DOI:** 10.7705/biomedica.6116

**Published:** 2022-06-01

**Authors:** Luis Fernando Valladales-Restrepo, Camilo Alexander Constain-Mosquera, María Alejandra Hoyos-Guapacha, Karol Liceth Hoyos-Guapacha, Andrés Gaviria-Mendoza, Manuel Enrique Machado-Duque, Jorge Enrique Machado-Alba

**Affiliations:** 1 Grupo de Investigación en Farmacoepidemiología y Farmacovigilancia, Universidad Tecnológica de Pereira-Audifarma S.A., Pereira, Colombia Universidad Tecnológica de Pereira Universidad Tecnológica de Pereira Audifarma S.A. Pereira Colombia; 2 Grupo de Investigación en Biomedicina, Facultad de Medicina, Fundación Universitaria Autónoma de las Américas, Pereira, Colombia Fundación Universitaria Autónoma de las Américas Fundación Universitaria Autónoma de las Américas Pereira Colombia; 3 Semillero de Investigación en Farmacología Geriátrica, Facultad de Medicina, Fundación Universitaria Autónoma de las Américas, Pereira, Colombia Fundación Universitaria Autónoma de las Américas Fundación Universitaria Autónoma de las Américas Pereira Colombia

**Keywords:** macrólidos, azitromicina, neumonía, prescripción inadecuada, infecciones por coronavirus, farmacoepidemiología, Colombia, Macrolides, azithromycin, pneumonia, inappropriate prescribing, coronavirus infections, pharmacoepidemiology, Colombia

## Abstract

**Introducción.:**

El uso inadecuado de antibióticos se asocia con aumento de la resistencia antimicrobiana, mayores costos de atención médica, más efectos adversos y peores resultados clínicos.

**Objetivo.:**

Determinar los patrones de prescripción y las indicaciones aprobadas y no aprobadas para macrólidos en un grupo de pacientes en Colombia.

**Materiales y métodos.:**

Se hizo un estudio de corte transversal sobre las indicaciones de uso de macrólidos en pacientes ambulatorios a partir de una base de datos de dispensación de medicamentos de 8,5 millones, aproximadamente, de personas afiliadas al sistema de salud de Colombia. Se consideraron variables sociodemográficas, farmacológicas y clínicas.

**Resultados.:**

Se encontraron 9.344 pacientes que habían recibido prescripción de macrólidos; su mediana de edad era de 40,1 años (rango intercuartílico: 27,1-53,3 años) y el 58,3 % correspondía a mujeres. El macrólido más prescrito fue la azitromicina (38,8 %) y los usos más frecuentes fueron el tratamiento de la infección por *Helicobacter pylori* (15,9 %) y la neumonía (15,8 %).

El 31,3 % de las prescripciones correspondía a indicaciones no aprobadas, destacándose el resfriado común (7,8 %), la Covid-19 (4,0 %) y la bronquitis aguda (3,5 %). La residencia en la región Caribe (OR=1,17; IC_95%_ 1,05-1,31), las prescripciones odontológicas (OR=2,75; IC_95%_ 1,91-3,96), las comorbilidades respiratorias crónicas (OR=1,30; IC_95%_ 1,08-1,56), y la prescripción de eritromicina (OR=3,66; IC_95%_ 3,24-4,14) o azitromicina (OR=2,15; IC_95%_ 1,92 2,41), se asociaron con una mayor probabilidad de recibir macrólidos para indicaciones no aprobadas, en tanto que tener entre 18 y 64 años (OR=0,81; IC_95%_ 0,71-0,93), 65 o más años (OR=0,77; IC_95%_ 0,63-0,94) y residir en Bogotá-Cundinamarca (OR=0,74; IC_95%_ 0,65 0,85) reducían dicho riesgo.

**Conclusiones.:**

La mayoría de los pacientes recibieron macrólidos para infecciones del sistema respiratorio; la eritromicina y la azitromicina se prescribieron en indicaciones no aprobadas en menores de 18 años y en quienes presentaban enfermedades respiratorias crónicas.

## Abstract (en)

**Introduction:**

The inappropriate use of antibiotics is associated with a greater risk for antimicrobial resistance, high health care costs, adverse events, and worse clinical outcomes.

**Objective:**

To determine the prescription patterns and approved and nonapproved indications for macrolides in a group of patients from Colombia.

**Materials and methods:**

This was a cross-sectional study on the indications for the use of macrolides in outpatients registered in a drug-dispensing database of approximately 8.5 million people affiliated with the Colombian health system. Sociodemographic, pharmacological, and clinical variables were considered.

**Results:**

A total of 9.344 patients had received a macrolide prescription; their median age was 40.1 years (interquartile range: 27.1-53.3 years) and 58.3% were women. The most commonly prescribed macrolide was azithromycin (38.8%) most frequently for *Helicobacter pylori* infection (15.9%) and pneumonia treatment (15.8%).

A total of 31.3% of the prescriptions were used for unapproved indications: common cold (7.8%), COVID-19 (4.0%), and acute bronchitis (3.5%). Residence in the Caribbean region (OR=1.17; 95%CI 1.05-1.31), dental prescriptions (OR=2.75; 95%CI 1.91-3.96), presence of chronic respiratory comorbidities (OR=1.30; 95%CI 1.08-1.56), and prescription of erythromycin (OR=3.66; 95%CI 3.24-4.14) or azithromycin (OR=2.15; 95%CI 1,92-2.41) were associated with a higher probability of macrolide use for unapproved indications while being 18-64 years old (OR=0.81; 95%CI 0.71-0.93) or 65 years or older (OR=0.77; 95%CI 0.63-0.94) and residing in Bogotá-Cundinamarca (OR=0.74; 95%CI 0.65-0.85) were associated with reduced risk.

**Conclusions:**

Most patients received macrolides for respiratory tract infections; erythromycin and azithromycin were used for unapproved indications in people under 18 years of age and those with chronic respiratory diseases.

La resistencia a los antibióticos es un problema creciente de salud pública a nivel mundial ^1^. El uso indiscriminado de este grupo de medicamentos ha crecido en más del 50 % en los últimos años ^1^, lo que ha resultado en un grave problema global de salud pública ^2^. La aceleración de la resistencia y la disminución del desarrollo de nuevos antibióticos para combatir este problema, implican importantes desafíos para los sistemas sanitarios, los profesionales de la salud y la población general en todo el mundo ^3^^,^^4^.

La Organización Mundial de la Salud (OMS) estimó que el 80 % de los antibióticos usados en los seres humanos se utiliza en la comunidad; de ese porcentaje, entre el 20 y el 50 % se emplea de forma inapropiada ^5^, ¿incluso en países de ingresos bajos y medios ^6^. El uso inadecuado de antibióticos se asocia con una mayor resistencia, incremento de costos de la atención médica, efectos adversos y peores resultados clínicos, incluidas complicaciones médicas, hospitalizaciones y muerte ^2^^,^^7^^,^^8^.

Los macrólidos se utilizan ampliamente y son el segundo grupo antibiótico más empleado en países como Estados Unidos ^9^ y Japón ^10^. En Colombia, su prescripción también es frecuente y se sitúa después de los betalactámicos, las fluoroquinolonas y las tetraciclinas ^11^. En general, este grupo de antibióticos es activo principalmente contra bacterias Gram positivas y bacterias atípicas, pero con acción limitada contra bacterias Gram negativas ^12^. La resistencia de los patógenos a los antibióticos se ha convertido en un problema terapéutico grave y persistente en la actualidad ^12^, por lo que fortalecer el conocimiento y el uso racional de los antibióticos entre los médicos de atención primaria es una forma de reducir dicha resistencia ^1^.

El sistema de salud de Colombia ofrece cobertura universal a la población por medio de dos regímenes de afiliación: el contributivo, que pagan conjuntamente trabajadores y empleadores, y el subsidiado, que cubre a las personas sin capacidad de pago y cuyo plan de beneficios incluye algunos antibióticos pertenecientes al grupo de los macrólidos. El objetivo de este estudio fue determinar sus patrones de prescripción y su uso en indicaciones aprobadas y no aprobadas en un grupo de pacientes de Colombia durante el 2020.

## Materiales y métodos

Se hizo un estudio de corte transversal para establecer la prescripción de macrólidos y su uso en indicaciones aprobadas y no aprobadas en pacientes ambulatorios registrados en una base de datos de dispensación de medicamentos que recoge información de 8,5 millones de personas, aproximadamente, afiliadas al sistema de salud por medio de seis compañías aseguradoras de salud, lo que corresponde, aproximadamente, al 30 % de la población activa afiliada al régimen contributivo o pago y al 6 % de la afiliada al régimen subsidiado por el Estado, que en total equivale al 17 % de la población colombiana.

Se detectaron los pacientes con una primera prescripción de macrólidos (eritromicina, claritromicina, azitromicina y espiramicina) entre el 1 de junio y el 30 de noviembre del 2020. Se seleccionaron individuos de cualquier sexo y edad atendidos en consulta médica ambulatoria por diagnósticos relacionados con infecciones listadas en la Clasificación Internacional de las Enfermedades, versión 10 (CIE-10). Se excluyeron los pacientes diagnosticados por razones diferentes a la infección.

A partir de la información sobre el uso de medicamentos de la población afiliada sistematizada por la empresa dispensadora (Audifarma S.A.), se diseñó una base de datos que reflejó los siguientes grupos de variables de los pacientes:

1. sociodemográficas, como edad, sexo, ciudad de dispensación y tipo de afiliación al sistema de salud de Colombia;
2. áreas geográficas, como lugar de residencia por departamentos y regiones de Colombia según la clasificación del Departamento Administrativo Nacional de Estadística (DANE): región Bogotá- Cundinamarca, región Caribe, región Central, región Oriental, región Pacífico y región Amazonía-Orinoquía;
3. comorbilidades crónicas, como principales enfermedades cardiovasculares, endocrinas, reumatológicas, urológicas, renales, psiquiátricas, neurológicas, digestivas, respiratorias y neoplásicas según los diagnósticos reportados con los códigos CIE-10;
4. farmacológicas, como macrólido prescrito (azitromicina, claritromicina, eritromicina o espiramicina), presentación farmacéutica (tableta, suspensión o solución para reconstituir en solución oral), dosis y duración del tratamiento utilizando las dosis diarias definidas como unidad de medida y según las recomendaciones de la OMS, expresada como dosis diaria definida por cada 1.000 habitantes por día ^13^;
5. tipo de prescriptor, como médico general, especialistas en medicina interna (pediatría, geriatría, fisiatría, etc.), especialistas en cirugía general, ortopedia, ginecoobstetricia, etc., odontólogos y profesionales no prescriptores (enfermería, optometría, nutrición o terapia respiratoria);
6. indicaciones, como diagnóstico principal y secundario asociado con cada prescripción de macrólidos según los códigos CIE-10, estableciendo si la indicación era aprobada o no aprobada según los registros de la *Food and Drug Administration* (FDA) de los Estados Unidos ^14^^-^^17^ y del Instituto Nacional de Vigilancia de Medicamentos y Alimentos (INVIMA) de Colombia ^18^.

Las indicaciones aprobadas fueron las siguientes.

- Azitromicina: exacerbación infecciosa aguda de la enfermedad pulmonar obstructiva crónica, otitis media aguda, conjuntivitis bacteriana, sinusitis bacteriana aguda, amigdalitis, faringitis estreptocócica, chancroide, infección por *Chlamydia trachomatis* (cervicitis - uretritis), enfermedad pélvica inflamatoria por *C. trachomatis*, *Neisseria gonorrhoeae* o micoplasma, neumonía, gonorrea e infección de piel o de tejidos subcutáneos no complicada. Además, el INVIMA lo indica en pacientes con enfermedad pulmonar obstructiva crónica (EPOC) y antecedentes de exacerbaciones recurrentes, a pesar del tratamiento con agonistas β2 adrenérgicos de larga acción, antimuscarínicos de larga acción y glucocorticoides inhalados ^18^.
- Claritromicina: exacerbación infecciosa aguda de la bronquitis crónica, otitis media aguda, neumonía adquirida en la comunidad, tratamiento o profilaxis de la infección diseminada debido al complejo *Mycobacterium avium-intracellulare*, erradicación de *Helicobacter pylori*, infección de piel o de tejidos subcutáneos no complicada, sinusitis aguda y faringitis estreptocócica.
- Eritromicina: acné, profilaxis de fiebre reumática en pacientes alérgicos a penicilina, profilaxis de endocarditis bacteriana, infección bacteriana ocular, infección por *Chlamydia trachomatis*, difteria, eritrasma, enfermedad pélvica inflamatoria, profilaxis neonatal de la conjuntivitis gonocócica o por clamidia, conjuntivitis por *Chlamydia trachomatis*, impétigo, infección leve a moderada de piel o tejidos subcutáneos, enfermedad del legionario, listeriosis, uretritis no gonocócica, tosferina, infección de vías respiratorias y sífilis.
- Espiramicina: sospecha o conocimiento de toxoplasmosis aguda en mujer embarazada durante las primeras 21 semanas de gestación y sin infección fetal.

El uso de macrólidos para el tratamiento de infecciones de piel y de tejidos blandos se consideró como una indicación no adecuada de acuerdo con las guías de práctica clínica del país ^19^.

El protocolo de estudio fue aprobado por el Comité de Bioética de la Universidad Tecnológica de Pereira en la categoría de investigación sin riesgo (código del aval: 01 - 23/11/20). Se respetaron los principios éticos establecidos por la Declaración de Helsinki.

Los datos se analizaron con el paquete estadístico SPSS Statistics, versión 26.0 para Windows (IBM, EE. UU.). Se hizo un análisis descriptivo con frecuencias y proporciones para las variables cualitativas, y medidas de tendencia central y de dispersión para las variables cuantitativas, dependiendo de su comportamiento paramétrico establecido mediante la prueba de Kolmogorov-Smirnov. Para la comparación de variables cuantitativas, se emplearon las pruebas t de Student y, para las categóricas, la de ji al cuadrado. Se desarrolló un modelo multivariado de regresión logística binaria que incluyó las variables asociadas en los análisis bivariados, así como aquellas con suficientes posibilidades o asociación reportada, con el fin de determinar las que se pudieran asociar con el uso de macrólidos en indicaciones no aprobadas después del ajuste. El nivel de significación estadística se fijó en p<0,05.

## Resultados

Se encontraron 9.344 pacientes de 160 ciudades diferentes que recibieron una primera prescripción de algún macrólido por diagnósticos relacionados con enfermedades infecciosas durante el periodo de estudio. El 58,3 % (n=5.447) correspondió a mujeres y la mediana de edad fue de 40,1 años (rango intercuartílico: 27,1-55,3 años; rango: 0,0-99,8 años). El 12,1 % (n=1.127) tenía menos de 18 años, el 37,6 % (n=3.512) se encontraba entre los 18 y 39 años, el 38,6 % (n=3.608) entre los 40 y 64 años y el 11,7 % (n=1.097) tenía 65 o más años.

Por regiones geográficas, los pacientes provenían principalmente de la región Caribe (n=4.374; 46,8 %), seguida de la de Bogotá-Cundinamarca (n=2.266; 24,3 %), la del Pacífico (n=1.242; 13,3 %), la Central (n=1.197; 12,8 %), la Oriental (n=219; 2,3 %) y la Amazonía-Orinoquía (n=46; 0,5 %). El 73,1 % (n=6.827) se encontraba afiliado al régimen contributivo y, el 26,9 % (n=2.517), al régimen subsidiado.

El 45,6 % (n=4.259) de los pacientes tenía alguna enfermedad crónica, con predominio de las cardiovasculares (n=2.034; 21,8 %), gastrointestinales (n=1.740; 18,6 %), endocrinas (n=1.114; 11,9 %), respiratorias (n=613; 6,6 %), psiquiátricas (n=296; 3,2 %), reumatológicas (n=288; 3,1 %), neurológicas (n=279; 3,0 %) y urológicas (n=151; 1,6 %). Las diez comorbilidades más comunes fueron hipertensión arterial (n=1.953; 20,9 %), gastritis crónica (n=1.452; 15,5 %), diabetes mellitus (n=487; 5,2 %), hipotiroidismo (n=337; 3,6 %), enfermedad pulmonar obstructiva crónica (n=333; 3,6 %), asma (n=290; 3,1 %), dislipidemia (n=232; 2,5 %), reflujo gastroesofágico (n=219; 2,3 %), síndrome de colon irritable (n=202; 2,2 %) y trastornos de ansiedad (n=177; 1,9 %).

El macrólido más prescrito fue azitromicina (n=3.629; 38,8 %), seguido de claritromicina (n=3.398; 36,4 %), eritromicina (n=2.059; 22,0 %) y espiramicina (n=258; 2,8 %) (cuadro 1). Las tabletas fueron las formas farmacéuticas más frecuentes (n=8.626; 92,3 %), seguidas del polvo para reconstituir en solución oral (n=451; 4,8 %) y las suspensiones (n=267; 2,9 %). Este grupo de antibióticos fue prescrito principalmente por médicos generales (n=8.609; 92,1 %), seguidos de profesionales de especialidades médicas (n=337; 3,6 %) o quirúrgicas (n=246; 2,6 %), y odontólogos (n=131; 1,4 %). El 0,2 % (n=21) de los pacientes recibió las prescripciones de personal no autorizado.

En el 76,5 % (n=7.161) de los pacientes, los macrólidos se utilizaron en el manejo de infecciones como gastritis aguda o infección por *H. pylori* (n=1.487; 15,9 %), neumonía (n=1.480; 15,8 %), amigdalitis aguda (n=926; 9,9 %), infecciones de las vías respiratorias no especificadas (n=766; 8,2 %), rinofaringitis aguda (n=730; 7,8 %), Covid-19 (n=376; 4,0 %), faringitis aguda (n=353; 3,8 %), bronquitis aguda (n=327; 3,5 %), otitis media aguda (n=247; 2,6 %), toxoplasmosis (n=237; 2,5 %) y sinusitis aguda (n=232; 2,5 %).

El 68,7% (n=6.422) de las prescripciones fueron para indicaciones aprobadas, en tanto que el 31,3 % (n=2.922) correspondió a indicaciones no aprobadas. La eritromicina fue el macrólido que se utilizó en mayor proporción para indicaciones no aprobadas (n=964-2.059; 46,8 %), seguido de azitromicina (n=1.275-3.629; 35,1 %), claritromicina (n=663-3398; 19,5 %) y espiramicina (20-258; 7,8 %). En el cuadro 2 se presentan las principales indicaciones no aprobadas.

**Cuadro 1:** Patrones de prescripción, frecuencia de uso, dosis medias, dosis diaria definida, distribución por sexo y edad en pacientes ambulatorios con dispensaciones de macrólidos, Colombia, 2020 (N=9.344)

Medicamento	n	%	Dosis prescrita (mg/día)				Sexo		Edad (años)		
			Media	Mediana	Moda	nDDD*	DHD	F (%)	M (%)	Media (DE)	Mediana (RIC)
Azitromicina	3.629	38,8	566,1	500	500	1,89	0,65	55,9	44,1	38,5 ± 19,2	36,8 ± 26,4
Claritromicina	3.398	36,4	1.000,0	1.000	1000	2,00	1,36	55,3	44,7	46,7 ± 18,9	47,4 ± 25,3
Eritromicina	2.059	22,0	1.463,1	1.500	1500	1,46	0,46	62,2	37,8	37,5 ± 20,6	36,5 ± 29,5
Espiromicina	258	2,8	2.895,3	3.000	3000	0,97	0,14	99,6	0,4	26,6 ± 6,0	26,0 ± 8,9

F: femenino; M: masculino; DE: desviación estándar; RIC: rango intercuartílico; DHD: dosis diaria definida por cada 1.000 habitantes por día

*Relación entre la dosis media prescrita y la dosis diaria definida

**Cuadro 2:** Indicaciones no aprobadas relacionados con la prescripción de macrólidos en un grupo de pacientes de Colombia, 2020 (N=9.344)

Diagnóstico	n	%
Rinofaringitis aguda (resfriado común)	730	7,8
Enfermedad del coronavirus 2019 (COVID-19)	376	4,0
Bronquitis aguda	327	3,5
Infecciones virales no especificadas	189	2,0
Absceso periapical	131	1,4
Periodontitis	129	1,4
Crisis asmática	116	1,2
Gastroenteritis	87	0,9
Celulitis o erisipela	76	0,8
Infección de vías urinarias	71	0,8
Vaginitis, vulvitis y vulvovaginitis	65	0,7
Otitis externa	61	0,7
Otros diagnósticos	564	6,0

**Cuadro 3:** Variables asociadas con usos no aprobados de macrólidos mediante una regresión logística binaria en pacientes ambulatorios, Colombia, 2020

Variables	Sig.	OR	IC_95%_	
			Inferior	Superior
Mujer	0,648	0,979	0,892	1,074
Edad <18 años	0,010	Referencia	Referencia	Referencia
Edad 18-64 años	0,004	0,815	0,710	0,936
Edad ≥65 años	0,011	0,775	0,637	0,944
Residir en la región Bogotá-Cundinamarca	<0,001	0,748	0,656	0,853
Residir en la región Caribe	0,004	1,178	1,054	1,316
Prescripción por odontología	<0,001	2,752	1,912	3,961
Comorbilidades cardiovasculares crónicas	0,152	1,092	0,968	1,231
Comorbilidades respiratorias crónicas	0,005	1,300	1,083	1,560
Prescripción de eritromicina	<0,001	3,668	3,245	4,147
Prescripción de azitromicina	<0,001	2,156	1,929	2,411

OR: *Odds Ratio*

IC95%: intervalo de confianza de 95 %

### Análisis multivariado

En la regresión logística binaria, se halló que la residencia en la región Caribe, la prescripción recibida de odontología, la prescripción de eritromicina o azitromicina y las comorbilidades respiratorias crónicas, se asociaron con una mayor probabilidad de que el macrólido se usara para indicaciones no aprobadas, en tanto que tener 18 o más años y residir en la región Bogotá- Cundinamarca redujeron este riesgo (cuadro 3).

## Discusión

El estudio permitió caracterizar el patrón de prescripción de antibióticos macrólidos para las indicaciones aprobadas y las no aprobadas, como evidencia del uso de medicamentos en el mundo real en un grupo de pacientes afiliados al sistema de salud de Colombia. Estos hallazgos pueden ser de utilidad para que, a la hora de las decisiones terapéuticas, el personal asistencial, académico y científico confronte los riesgos de los pacientes, así como para fortalecer las prácticas de uso adecuado de los antibióticos entre los médicos como una forma de reducir la resistencia bacteriana en el país.

Entre los diferentes antibióticos de este grupo farmacológico, predominó la prescripción de azitromicina, lo que coincide con publicaciones previas en Colombia (41,0 %) ^11^, y en pacientes ambulatorios de otros países como México (57,7 %) ^20^ y Ecuador (55,7 %) ^21^, pero contrasta con lo hallado en Malasia, en donde prevaleció el uso de la eritromicina ^22^, en tanto que en Siria fue mayor el uso de la claritromicina ^23^. Al considerar estos patrones de uso durante la pandemia de Covid-19, en diferentes estudios se ha informado un mayor empleo de azitromicina ^24^^,^^25^, lo que concuerda con los hallazgos acá registrados. Las dosis diarias definidas por 1.000 personas por día es la medida preferida para comparar el consumo de medicamentos entre países, y comprender mejor los patrones y cantidades utilizadas a nivel nacional, de manera que puedan hacerse intervenciones que optimicen el uso de los antibióticos ^13^. En este sentido, Adriaenssens, *et al*., presentaron datos de la Red Europea de Vigilancia del Consumo de Antimicrobianos (ESAC-Net) sobre los macrólidos empleados ambulatoriamente en 30 países de la Unión Europea entre 1997 y 2017. Al comparar los hallazgos del último año con los de nuestro análisis, se encontró que, en la gran mayoría de países (excepto en Reino Unido y Noruega), las dosis diarias definidas por 1.000 personas por día de la eritromicina fueron menores, en tanto que, casi en la mitad de los países, las de azitromicina y claritromicina fueron mayores ^26^.

También como consecuencia de la pandemia de Covid-19, es posible que estos patrones de uso de macrólidos presenten variaciones con respecto al periodo prepandemia, como se ha documentado en Jordania, en donde las dosis diarias definidas por 1.000 personas por día de los macrólidos pasó de 3,23 en el 2019 a 4,81 en el 2020, con un incremento del 74 % para la azitromicina ^27^. Sin embargo, en otros reportes de Reino Unido y Estados Unidos, se evidenció una reducción en el uso de antibióticos, entre ellos, los macrólidos, en los meses correspondientes al confinamiento ^25^^,^^28^^,^^29^. Además de los factores relacionados con la Covid-19, la variación en la dispensación de estos medicamentos depende de diversos factores, como el tipo de formación académica del médico, la autorización y disponibilidad de los medicamentos en cada país, sus indicaciones, patrones de sensibilidad y resistencia locales, sus costos, las características del paciente (antecedente de hipersensibilidad), así como del cubrimiento de los medicamentos en el plan de beneficios en salud ^14^^-^^18^^,^^26^^,^^27^^,^^30^.

En este sentido, en el 2020 todas las presentaciones de la azitromicina y las formas farmacéuticas orales convencionales de la claritromicina, se encontraban cubiertas por el plan de beneficios en salud de Colombia solo para los pacientes con diagnóstico de neumonía, en tanto que no había restricciones para la prescripción de eritromicina y espiramicina ^30^.

Los macrólidos se emplearon principalmente en los esquemas de erradicación de la infección por *H. pylori*, lo que coincide con otros estudios ^31^^-^^33^. Según las guías internacionales y locales, la claritromicina asociada con uno o dos antibióticos de otros grupos farmacológicos, junto con un inhibidor de la bomba de protones con o sin sales de bismuto, conforman los esquemas de manejo eficaces; sin embargo, la resistencia a la claritromicina ha ido aumentando progresivamente en diferentes regiones del mundo, lo que puede llevar al fracaso del tratamiento, por lo que se recomienda su uso cuando la resistencia sea inferior al 15 % ^34^^,^^35^.

Otra de las principales indicaciones de este grupo farmacológico fue el tratamiento de la neumonía, lo que contrasta con lo reportado en otros estudios en los que se empleó principalmente para infecciones de las vías respiratorias altas ^10^^,^^36^, como sinusitis (18,0 %) ^36^ y faringitis (12,7 %) ^10^. En la guía de tratamiento de la neumonía adquirida en la comunidad para adultos inmunocompetentes del país, se recomiendan los macrólidos en la monoterapia para el tratamiento ambulatorio de pacientes con neumonía sin factores de riesgo, y asociado con un betalactámico cuando estos factores están presentes, ya sea en el ámbito ambulatorio o el hospitalario ^37^, recomendaciones que están avaladas también a nivel internacional ^38^. Asimismo, este grupo farmacológico se emplea para la neumonía en edades pediátricas ^39^.

El 31,3 % de las prescripciones de macrólidos se usaron en indicaciones no aprobadas, lo cual resulta inferior a lo reportado en otros países ^1^^,^^36^^,^^40^^,^^41^, por ejemplo en Qatar, donde la prescripción inadecuada de macrólidos fue de 52,0 % ^7^, en Estados Unidos, donde fluctuó entre 52,0 y 78,4 % ^36^^,^^40^, en China, donde fue de 84,2 % ^1^, y en Pakistán, donde en pacientes hospitalizados fue de 62,7 % ^41^. Coincide, sin embargo, con lo reportado por Calderón-Parra, *et al*., en España en pacientes con Covid-19, en quienes el uso de antibióticos fue inapropiado en un 34,2 % ^42^. Esta problemática no compete solamente a los macrólidos, pues en general se ha descrito con todos los antibióticos ^21^^,^^43^. En Colombia, se ha evidenciado que aproximadamente una cuarta parte de las prescripciones de fluoroquinolonas y tetraciclinas se emitieron para indicaciones no aprobadas ^44^^,^^45^.

La mayoría de los usos no aprobados de los macrólidos involucraron enfermedades de las vías respiratorias superiores, como lo documentan otros estudios ^7^^,^^46^, en los que se halló que el principal uso no aprobado de los macrólidos fue la rinofaringitis aguda (resfriado común), problema que también se ha evidenciado en otros países ^10^^,^^22^^,^^36^^,^^43^ con otros antibióticos, como los betalactámicos y las fluoroquinolonas ^44^^,^^46^. Para esta condición, no se recomienda ningún antibiótico, puesto que su etiología es viral ^47^. Asimismo, el uso de macrólidos para la bronquitis aguda ha sido frecuente, pero inferior a lo hallado en Estados Unidos (14,0 %) ^36^, Japón (10,6 %) ^10^ e Italia (5,3 %) ^43^. El uso innecesario de antibióticos en infecciones de etiología viral debe responderse con intervenciones para incentivar su uso adecuado, en especial cuando la condición más común asociada con la excesiva prescripción de antibióticos es la infección de las vías respiratorias superiores ^2^.

Debe anotarse que el 4,0 % de las prescripciones de macrólidos (azitromicina) fue para pacientes con Covid-19. Su uso en este contexto clínico tal vez tenga sus orígenes en un estudio francés publicado a inicios del 2020; según sus autores, el tratamiento con hidroxicloroquina se asociaba con la reducción significativa de la carga viral en estos pacientes y su efecto aumentaba al adicionar azitromicina ^48^, lo cual fue difundido ampliamente por los medios de comunicación, y llevó a su rápida y amplia utilización en diversos países; sin embargo, hasta el momento no se han demostrado los efectos beneficiosos de los macrólidos comparados con el tratamiento estándar ^49^. El consenso colombiano de atención, diagnóstico y manejo de la infección por SARS-COV-2/Covid-19, desaconseja el uso de la azitromicina como antiviral ^50^, pero a pesar de la evidencia y de las guías de manejo actualizadas, se sigue utilizando en este grupo de pacientes tal como se evidenció en nuestro estudio.

Por otro lado, las enfermedades más frecuentes en los pacientes que recibieron prescripciones de estos antibióticos fueron los trastornos cardiovasculares, destacándose entre ellos la hipertensión arterial sistémica, lo que concuerda con lo hallado en otro grupo de pacientes tratados con antibióticos en Colombia ^11^. Los macrólidos se han asociado con la prolongación del intervalo QT y, por lo tanto, con un mayor riesgo de arritmias, como fibrilación auricular, taquicardia supraventricular, torcida de puntas, taquicardia y fibrilación ventricular, entre otras ^51^, cuya aparición puede aumentar con el uso concomitante de un grupo heterogéneo de medicamentos, en mayores de 65 años y en quienes tienen comorbilidades como hipertensión arterial, cardiopatía isquémica o falla cardiaca, entre otros ^52^. Por ello, la prescripción de macrólidos debe hacerse con mucha precaución, sopesando el beneficio y los riesgos de instaurar ese tratamiento antimicrobiano.

Según lo hallado en otros estudios, el sexo no se relacionó con la probabilidad de tener prescripciones no aprobadas de macrólidos ^1^^,^^8^, en tanto que los pacientes mayores de 18 años tuvieron un menor riesgo (19 23 %), lo cual contrasta con el estudio de Chang, *et al*., en China, donde este grupo de pacientes hospitalizados tuvo una mayor probabilidad (20-36 %) ^1^. Por otra parte, tener enfermedades respiratorias crónicas elevó la probabilidad de formular recibir una fórmula de un macrólido para un uso no aprobado, debido a que estos pacientes pueden presentar exacerbaciones de la condición de base que se tratan con antibióticos, pasando por alto que tales agudizaciones no siempre son de origen bacteriano, y sí de etiología viral o no infecciosa ^53^^,^^54^.

Por último, la prescripción de macrólidos por parte de profesionales de la odontología también elevó la probabilidad de su uso inadecuado, ya que no se recomienda emplearlos en presencia de abscesos periapicales y periodontitis, prefiriéndose las aminopenicilinas, con o sin inhibidores de la betalactamasa, la fenoximetilpenicilina, el metronidazol o la clindamicina, ya que tienen una mayor actividad contra los microorganismos implicados en las infecciones de la cavidad bucal ^55^^-^^57^. Sin embargo, según el perfil de resistencia local, los macrólidos se podrían emplear como alternativa en pacientes con alergia a las penicilinas ^56^.

Debemos señalar algunas limitaciones en la interpretación de los resultados, puesto que no se obtuvo acceso a las historias clínicas para verificar las enfermedades de los pacientes y, así, confrontar la precisión de los diagnósticos asignados por el médico. Además, solo fue posible establecer el diagnóstico principal asociado con cada dispensación y no se estableció cuáles pacientes presentaban exacerbaciones recurrentes de enfermedad pulmonar obstructiva crónica. También, se desconocen los medicamentos formulados por fuera del sistema de salud, o que no son entregados por la empresa dispensadora y que los pacientes pudieron haber recibido. Cabe señalar que los profesionales de salud diferentes a los médicos a cargo de las prescripciones, son parte del personal autorizado por los aseguradores para refrendar fórmulas en la mayor parte del territorio nacional, tanto en el régimen contributivo como en el subsidiado.

Con base en estos hallazgos, se puede concluir que más de la mitad de los pacientes del estudio recibieron macrólidos para infecciones de las vías respiratorias inferiores y superiores, y que fue menor su uso para la infección por *H. pylori* e, incluso, para la Covid-19; por ote parte, una tercera parte de los casos correspondió a indicaciones no aprobadas por las agencias reguladoras, en particular, el uso de eritromicina y azitromicina en menores de 18 años y en quienes presentaan enfermedades respiratorias crónicas. Estos hallazgos pueden ser útiles para los médicos que tratan infecciones, para los responsables de las decisiones y para los prescriptores.
